# Genetic dissection of yield and yield-related traits in mungbean based on QTL meta-analysis

**DOI:** 10.3389/fgene.2025.1600979

**Published:** 2025-05-08

**Authors:** Binbin Du, Qifei Wang, Song Gao, Fan Yang, Lei Zhang, Xiao Zhang, Dongao Huo, Jia Wu, Xingen Zhang, Fang Li, Baowei Lu, Fengxia An

**Affiliations:** ^1^ College of Biotechnology and Pharmaceutical Engineering, West Anhui University, Lu’an, Anhui, China; ^2^ Institute of Crop and Nuclear Technology Utilization, Zhejiang Academy of Agricultural Sciences, Hangzhou, Zhejiang, China; ^3^ College of Biological Sciences and Technology, Taiyuan Normal University, Taiyuan, Shanxi, China

**Keywords:** mungbean, yield, QTL meta-analysis, meta-QTL, candidate gene

## Abstract

As an important food crop and nutritional source, mungbean has prioritized yield improvement as a key objective in breeding programs. In present study, we conducted a QTL meta-analysis to integrate 660 QTLs related to yield and yield-related traits in mungbean published over the past 20 years. A total of 590 initial QTLs were mapped onto a high-density consensus map, resulting in the identification of 72 meta-QTLs (MQTLs). These MQTLs were unevenly distributed across 11 linkage groups (LGs) with an average confidence interval (CI) of 1.21 cM, which was 6.26-fold narrower than the average CI of the initial QTLs. Among these 72 MQTLs, 20 were validated in a genome-wide association study (GWAS) for yield and yield-related traits in mungbean. Orthologous MQTL analysis revealed that 22 mungbean MQTLs were collinear with 19 MQTLs in common bean for yield and yield-related traits. In addition, 20 breeder’s MQTLs were screened from the 72 MQTLs, and 339 gene models were identified within the breeder’s MQTL regions. Twenty-two mungbean orthologs of yield-related genes such as seed germination, tiller number, and plant height in rice and *Arabidopsis* were identified in the breeder’s MQTL regions using homology analysis. This study contributes to understanding the genetic mechanisms for yield and yield-related traits and provides new ideas for the genetic improvement and breeding of mungbean.

## Introduction

Mungbean (*Vigna radiata* L.), as one of the most important legumes, provides a vital source of nutrition for human beings due to its high protein and carbohydrate content (Somta et al., 2007). Additionally, mungbean is widely cultivated for both food and medicinal purposes, owing to its health benefits such as clearing heat and toxins and quenching thirst (Tang et al., 2014). The low yield of mungbean, with an average grain yield of only about 700 kg per hectare (Islam et al., 2014), coupled with its lower economic returns compared to traditional crops, has led to a gradual decline in its cultivation area. This yield stagnation is exacerbated by global challenges such as insect pests, diseases (e.g., mungbean yellow mosaic virus and bruchid infestations), and environmental stresses including drought and soil salinity, which disproportionately affect smallholder farmers in Asia and Africa (Pandey et al., 2018). This trend has seriously affected the development of the mungbean cultivation industry in China and other major producing countries like India and Myanmar, where climate variability further threatens production stability (Nair et al., 2019). Therefore, improving yield remains a key objective of mungbean breeding (Nair and Schreinemachers, 2020). In mungbean, grain yield is influenced not only by the number of pods per plant, number of grains per pod, and the 100-grain weight traits, but also by plant height, number of branches, pod morphology, leaf morphology, and fertility period are also important factors affecting mungbean yield (Ahmad and Belwal, 2020).

Understanding the genetic basis of yield and yield-related traits is essential for genetic improvement and achieving breeding goals in mungbean. To date, several studies have employed linkage analysis in mungbean to identify quantitative trait loci (QTL) for yield-related traits such as 100-grain weight, pod length, plant height, and grain morphology (Humphry et al., 2005; Isemura et al., 2012; Liu et al., 2017; Ye et al., 2021; Vu et al., 2022). Nevertheless, the results of QTL linkage analyses based on segregating populations are susceptible to various factors, including the types and densities of markers used to construct genetic maps, parental selection, the types and sizes of mapping populations, the experimental environments, and differences in statistical analysis methods (Zhang et al., 2017). Notably, although numerous QTLs can be identified in segregating populations, most of them are minor-effect QTLs with relatively low stability and reliability (Brachi et al., 2010), which limits their application in molecular-assisted selection (MAS) and gene cloning.

In addition to QTL linkage analysis, genome-wide association studies (GWAS) have also been employed as a reliable technique for identifying candidate genes associated with complex quantitative traits in mungbean (Breria et al., 2020; Liu J. H. et al., 2022; Chang et al., 2023; Manjunatha et al., 2023). The mutual validation of quantitative traits using both QTL linkage analysis and GWAS has led to the discovery of key loci for the target trait in several studies (Zhang et al., 2019; Song et al., 2020; Wei et al., 2021), such as the identification of QTrl.saw-2D.2, an important QTL controlling root length, through linkage and association analyses in wheat (Zheng et al., 2019). This indicates that combining QTL linkage analysis and GWAS results is beneficial in identifying key genomic regions for important yield traits in mungbean.

QTL meta-analysis is a statistical genetics approach integrating multiple QTL datasets via a consensus genetic map, which integrates QTL results from different mapping populations, traits, and environments through statistical methods to identify the consistency and validity of the QTLs to obtain meta-QTLs (MQTL) (Goffinet and Gerber, 2000; Sosnowski et al., 2012). In recent years, QTL meta-analysis has been applied to multiple crops for different quantitative traits, such as grain quality traits, flag leaf morphology and yield-related traits in wheat (Yang et al., 2021; Du et al., 2022; Gudi et al., 2022; Saini et al., 2022; Vasistha et al., 2024), grain weight, resistance, and yield-related traits in rice (Khahani et al., 2020; Li et al., 2020; Anilkumar et al., 2022), popping traits, root-related traits and grain yield traits in maize (Pan et al., 2017; Guo et al., 2018; Kumar et al., 2021), and grain quality traits, resistance and yield-related traits in barley (Akbari et al., 2022; Du et al., 2024a; Du et al., 2024b). Meanwhile, QTL meta-analysis was performed for certain agronomic traits in legume crops, such as grain quality traits in soybean (Chen et al., 2021) and grain quality traits and yield-related traits in pigeonpea (Halladakeri et al., 2023). However, QTL meta-analysis for mungbean has not yet been reported. This study represents the first effort to integrate QTL data from various studies to identify MQTL for yield and yield-related traits in mungbean.

In this study, a QTL meta-analysis was conducted based on 18 QTL studies focused on yield and yield-related traits in mungbean to identify MQTLs and candidate genes associated with yield and yield-related traits such as 100-grain weight, plant height, grain morphology, fertility, and pod morphology. The main objectives of this study were (i) to identify stable and reliable MQTLs for yield and yield-related traits in mungbean; (ii) to compare GWAS results for yield and yield-related traits in mungbean with MQTLs; (iii) to identify orthologous MQTL by comparing synteny and colinearity among mungbean, soybean, and pigeonpea; and (iv) to identify prospective candidate genes within the breeder’s MQTL regions.

## Materials and methods

### Construction of consensus genetic maps and QTL meta-analysis

A high-density consensus genetic map was generated by integrating seven reference genetic maps using the R package LPmerge (Endelman and Plomion, 2014). The detailed LPmerge code is listed in Supplementary Data S1. The integrated maps included: (i) “Mungbean-Berken × ACC41-RIL” (Humphry et al., 2005); (ii) “Mungbean-JP211874 × JP229096-BC_1_F_1_” (Isemura et al., 2012); (iii) “Mungbean-Berken × ACC41-RIL” (Wu et al., 2014); (iv) “Mungbean-VC2917 × ZL-RIL” (Liu et al., 2017); (v) “Mungbean-Huaye1 × Zijing1-F_2_” (Wang et al., 2017); (vi) “Mungbean-Dahuaye × Jilv9-RIL” (Wang et al., 2020); (vii) “Mungbean-Sulu16-10 × Weilu11-F_2_” (Ye et al., 2021).

Following map integration, the consensus map and initial QTL data were imported into BioMercator V4.2.3 for analysis (Supplementary Table S1). Initial QTLs were projected onto the consensus map using the QTLProj module (Veyrieras et al., 2007). QTL meta-analysis was performed via the Veyrieras two-step method to identify MQTLs (Sosnowski et al., 2012). Firstly, QTL clustering was performed on each chromosome using BioMercator’s standard parameters. The optimal number of MQTLs was determined by comparing five statistical criteria: Akaike information criterion (AIC), AIC correction, AIC3, Bayesian information criterion (BIC), and Average Weight of Evidence (AWE). The model with the frequent values across all criteria was selected. Second, Second, the 95% confidence interval (CI) and peak position of each MQTL were defined based on the best-fit model from the first stage. Initial QTLs falling within the MQTL CI were integrated, while those failing to meet the minimum AIC threshold were excluded (Aloryi et al., 2024). Information on the files corresponding to the first step (_model.txt) and the second step (_table.txt) is provided in Supplementary Data S2. MQTLs were systematically named according to their chromosomal locations (e.g., MQTLLG1-1, MQTLLG1-2). The phenotypic variance explained (PVE) by each MQTL was calculated as the mean PVE of its constituent QTLs.

### Data collection of QTL for yield and yield-related traits

Literature searches were conducted across databases including Google Scholar (https://scholar.google.com/), PubMed (https://pubmed.ncbi.nlm.nih.gov/), and CNKI (https://www.cnki.net/) for the systematic search for QTL studies on yield and yield-related traits in mungbean published from 2005 to present. From these studies, we extracted the following parameters: parental composition of the population, population type and size, measured traits, molecular marker types, QTL flanking markers, logarithm of odds (LOD) scores, phenotypic variance explained (PVE) or *R* ^2^, and confidence interval (CI) (Supplementary Tables S2, S3). QTLs lacking reported LOD scores were assigned a default value of 3, while those with missing PVE values were excluded. For several QTLs with missing CI information, CI (95%) was calculated using population-specific formulas (Darvasi and Soller, 1997; Guo et al., 2006).

(1) $\text{CI}=287\;/\;\left(n\;\times \text{ PVE}\right)\text{ for DH populations}$

(2) $\text{CI}=163\;/\;\left(n\;\times \text{ PVE}\right)\text{ for the RIL population}$

(3) $\text{CI}=530\;/\;\left(n\;\times \text{ PVE}\right)\text{ for }F2\text{ and BC populations}$

These initial QTLs were associated with 71 different traits, categorized into nine groups: (i) growth period-related traits (22 traits such as branching stage, days to flowering, days to harvest, and days to maturity); (ii) yield traits (yield per block, yield index, yield per plant, biomass index and biomass); (iii) branch number (branch number per plant and number of branches); (iv) leaf related traits (chlorophyll content, leaf width, maximum leaf area, and other 8 traits); (v) hundred-grain weight; (vi) plant height and plant height related traits (plant height, number of nods, main stem length, plant height index and stem internode length); (vii) pod related traits (pod number per plant, total number of pods, pod length and other 7 traits); (viii) seed related traits (hard seedness, seed diameter, seed length and other 9 traits); (ix) other traits (12 traits such as main stem thickness, growth habit, germinating percentage) (Supplementary Table S4).

### Physical mapping and GWAS validation of MQTLs

To determine the physical location of these MQTLs, we performed BLASTn alignment of MQTL flanking marker sequences against the *V. radiata* L. reference genome (Kang et al., 2014) available via EnsemblPlants (https://plants.ensembl.org/). However, due to insufficient sequence information for most flanking markers, direct physical localization was unfeasible. We therefore calculated physical locations using the following genetic-to-physical conversion formula (Prakash et al., 2022):

$$\text{Physical location }\left(\text{bp}\right)=\frac{\text{Chromosome}\;\text{physical}\;\text{length}\;\left(\text{bp}\right)}{\text{chromosome}\;g\text{enetic}\;\text{length}\;\left(\text{cM}\right)}\times \text{Genetic location }\left(\text{cM}\right)$$

Chromosomal physical lengths were obtained from the mungbean reference genome, while genetic lengths were derived from our consensus map constructed. For GWAS validation, we compiled marker-trait associations (MTAs) from six GWAS studies associated with yield and yield-related traits in mungbean (published in 2018–2023), recording population size, traits, marker types, and MTA counts (Table 2). MQTLs were considered validated if their physical intervals overlapped with ≥1 MTA.

### Orthologous MQTL analysis among legume crops

To identify the OrMQTLs for yield and yield-related traits in mungbean, pigeonpea, and common bean, the following steps were taken: (i) identify conserved regions in the genomes of mungbean, pigeonpea, and common bean using synteny and colinearity analyses with the ‘BioMart’ tool in the EnsemblPlants database (https://plants.ensembl.org/biomart/); (ii) screen for pigeonpea and common bean orthologs within the region of the MQTL in mungbean genomes; (iii) compare the physical positions of the pigeonpea and common bean orthologs to the MQTL regions of the corresponding yield and yield-related traits (Halladakeri et al., 2023; Izquierdo et al., 2023), and consider the MQTLs of pigeonpea and common bean that contain at least four of the corresponding genes as OrMQTLs for mungbean. The synteny analysis between mungbean, pigeonpea, and common bean genomes was plotted using Tbtools software (Chen et al., 2020).

### Candidate gene mining within breeder’s MQTL regions

According to the criteria developed by Löffler et al. (2009), MQTLs with genetic distances <2 cM, containing at least four initial QTLs from different studies, and with PVE >10% were screened as breeder’s MQTLs for candidate gene mining. The information of gene models within the breeder’s MQTL regions was searched using the EnsemblPlants database (https://plants.ensembl.org/). To identify candidate genes, a comparative genomics approach was implemented to mine orthologs of yield-related genes from rice and *Arabidopsis thaliana* within the breeder’s MQTL regions of the mungbean genome.

## Results

### Consensus genetic map

**FIGURE 1:**
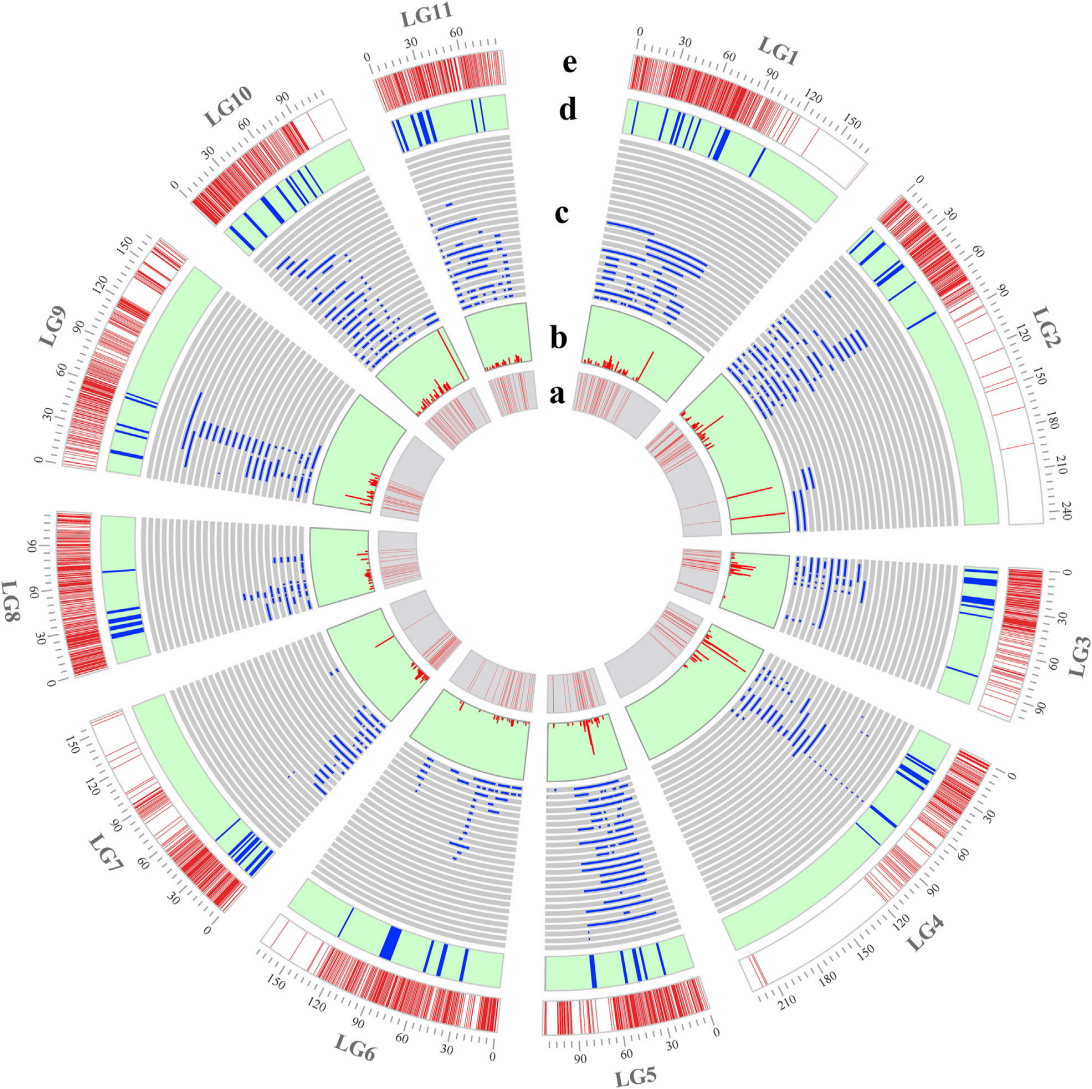
Position of initial QTLs and MQTLs on the consensus genetic map. **(a)** Density of initial QTLs: regions with low or high marker densities are indicated in light and dark red, respectively; **(b)** PVE value of each initial QTL; **(c)** position of initial QTLs on the consensus genetic map; **(d)** position of MQTLs on the consensus genetic map; and **(e)** density of molecular markers per linkage group.

A high-density consensus genetic map was constructed using the R package LPmerge to integrate seven previously published genetic maps in mungbean. The consensus genetic map spanned a genetic distance of 1,679.58 cM and contained 3,497 markers, with an average genetic distance between markers of 0.48 cM (Table 1; Supplementary Table S1). The genetic length of individual linkage groups ranged from 87.80 cM (LG11) to 246.60 cM (LG2), with an average length of 152.69 cM across all linkage groups. The number of markers per linkage group varied from 212 (LG11) to 426 (LG1), with an average of 317.91 markers per linkage group. The marker density of individual linkage groups ranged from 1.40 (LG4) to 3.32 (LG5) markers per cM, with an overall average density of 2.22 markers per cM (Figure 1e; Table 1).

**TABLE 1:** Detailed information on the high-density consensus genetic map in mungbean

Linkage group	Markers (no.)	Length (cM)	Marker density
LG1	426	174.70	2.44
LG2	371	246.60	1.50
LG3	245	104.10	2.35
LG4	317	225.90	1.40
LG5	376	113.37	3.32
LG6	369	171.60	2.15
LG7	312	158.08	1.97
LG8	346	112.09	3.09
LG9	285	168.03	1.70
LG10	238	117.30	2.03
LG11	212	87.80	2.41
Average	317.91	152.69	2.22

### Characterisation of initial QTL for yield and yield-related traits

**FIGURE 2:**
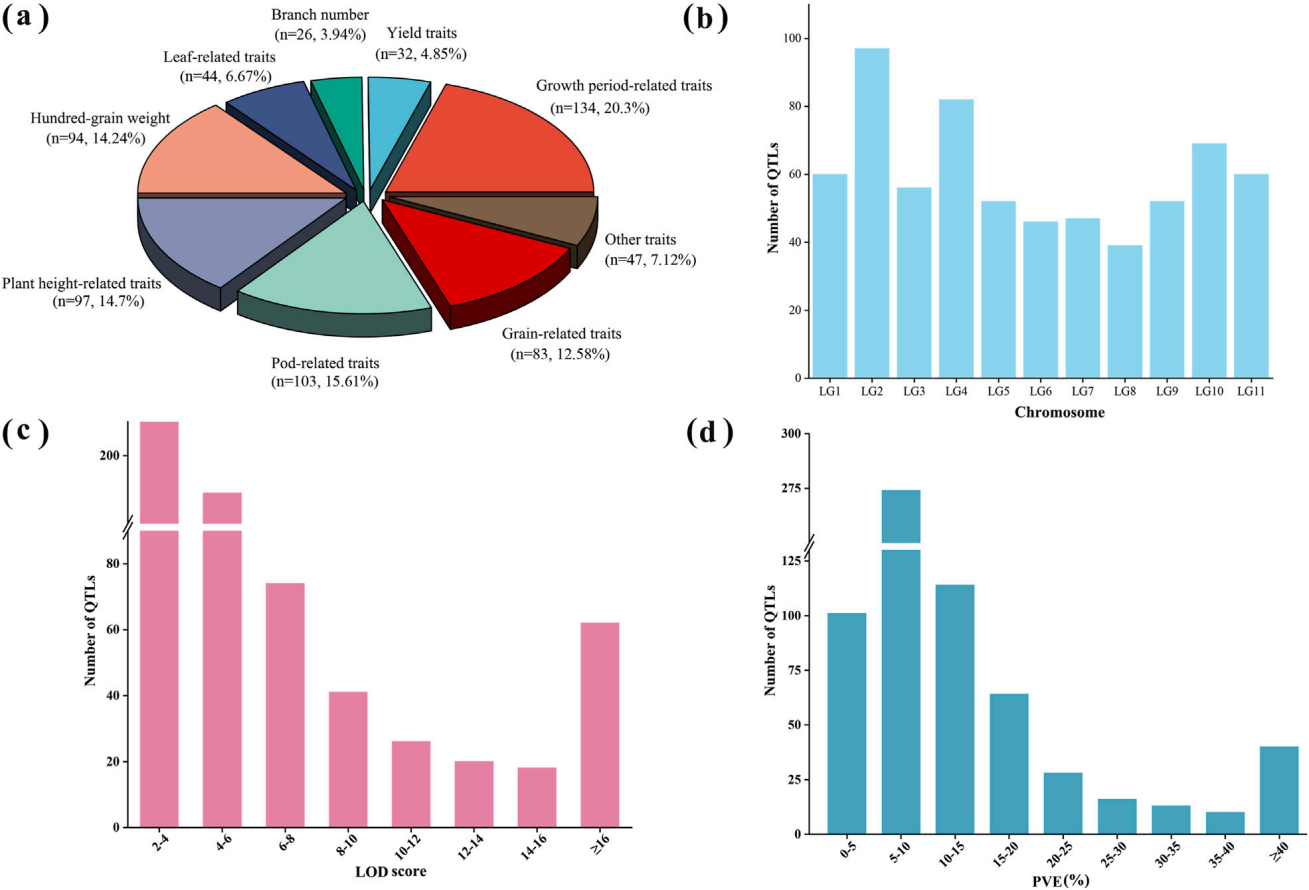
Information on initial QTLs for yield and yield-related traits in mungbean. **(a)** Percentage of initial QTLs for different types of traits; **(b)** Distribution of initial QTLs on linkage groups; **(c)** Frequency distribution of LOD scores of initial QTLs; **(d)** Frequency distribution of PVE (%) of initial QTLs.

A total of 660 QTLs from 18 independent studies (2005–2023) involving QTL mapping for yield and yield-related traits in mungbean were collected during 2005–2023 for meta-analysis (Supplementary Tables S2, S3). These studies included a total of 21 different QTL mapping populations, including 12 RIL populations, 6 F_2_ populations, 2 F_3_ populations, and 1 backcross population, with population sizes ranging from 100 to 261 (Supplementary Table S2). The yield and yield-related traits analyzed were categorized into nine types (Supplementary Table S4). Among these, the number of QTLs associated with growth period-related traits, pod-related traits, plant height, hundred-grain weight and grain-related traits was relatively high, accounting for 20.3%, 15.61%, 14.7%, 14.24% and 12.58% of the total number of QTLs, respectively, while the remaining traits represented a smaller proportion (Figure 2a). These QTLs were unevenly distributed across the linkage groups, with LG2 containing the highest number of QTL at 14.70% (97/660), LG8 having the lowest at 5.90% (39/660), and the remaining linkage groups ranging from 46 to 82 QTLs each (Figure 1a,c; Figure 2b). The logarithm of odds (LOD) scores for these QTLs ranged from 2 to 97.25, with the majority (63.6%) falling between 2 and 6 (Figure 2c). The phenotypic variance explained (PVE) by individual QTLs ranged from 1.32% to 99.51%, with an average value of 14.2%, and 41.5% were distributed within 5%–10% range (Figure 1b; Figure 2d).

### MQTL analysis for yield and yield-related traits

**FIGURE 3:**
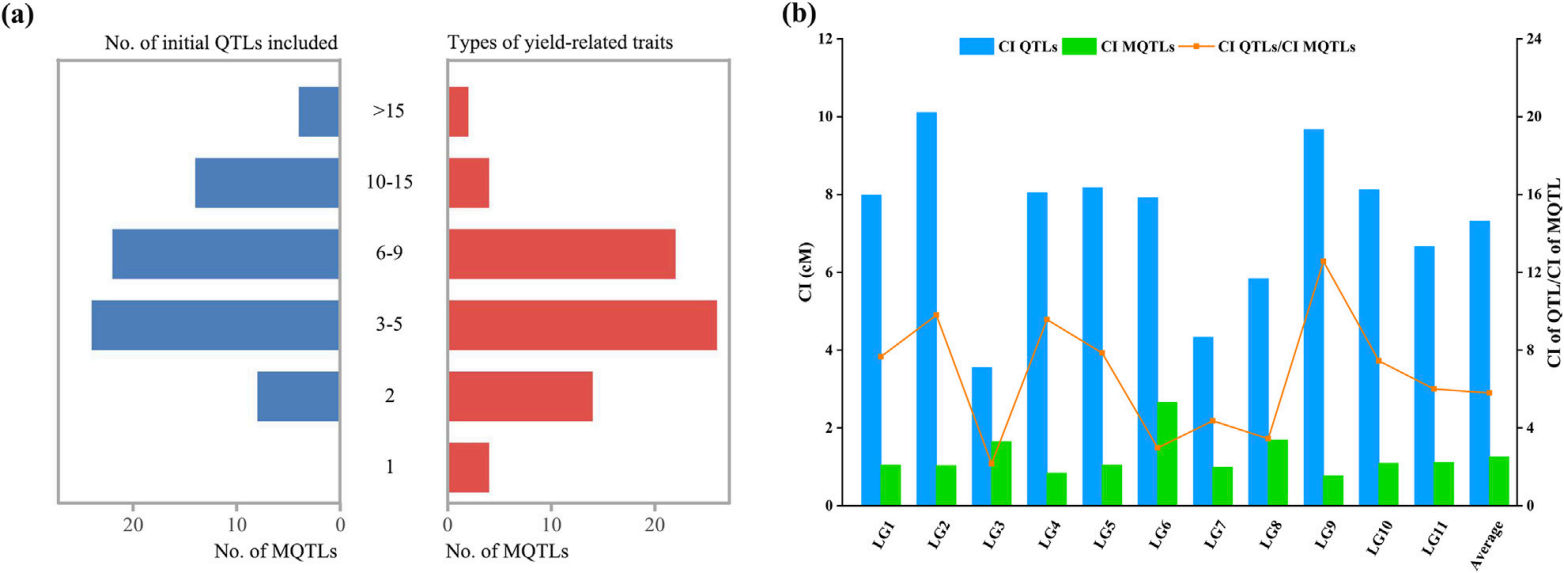
Basic information of the MQTLs. **(a)** Number of MQTLs containing the number of initial QTLs (left) and the number of MQTLs containing the number of different yield-related traits (right); **(b)** Comparison of confidence intervals between the initial QTLs (blue bars) and the MQTLs (cyan bars); The orange line represents the reduced fold of the QTL confidence interval.

Among the 660 initial QTLs for yield and yield-related traits, 590 were screened for mapping onto the consensus genetic map after excluding QTLs with missing PVE values or flanking markers for meta-analysis. Of these 590 initial QTLs, meta-analysis integrated 553 QTLs into 72 MQTLs, while 37 QTLs remained as individual QTLs without overlapping any MQTL (Supplementary Table S5). The MQTLs were unevenly distributed across the linkage groups, with the number of MQTLs ranging from 5 in LG6, LG8, and LG9 to 9 in LG1 (Figure 1d). Each MQTL contained at least 2 initial QTLs, and 64 MQTLs consisted of no fewer than 3 initial QTLs, with *MQTLLG4-6* comprising up to 46 initial QTLs (Figure 3a; Supplementary Table S5). Among the 72 MQTLs, 75% (54/72) were associated with at least three yield and yield-related traits, and *MQTLLG2-5* and *MQTLLG4-6* affected 18 yield and yield-related traits simultaneously (Figure 3a; Supplementary Table S5). The confidence intervals (CIs) of these MQTLs ranged from 0.09 to 8.28 cM, with an average CI of 1.21 cM, representing a 6.26-fold reduction compared to the average CI of the initial QTL. The reduction in CI varied significantly across all linkage groups, with the largest reduction observed in LG9 (12.59-fold) and the smallest in LG3 (2.16-fold) (Figure 3b).

### GWAS-MTA verification MQTLs

A total of 5,563 marker-trait associations (MTAs) were identified from six GWAS studies on yield and yield-related traits in mungbean published since 2018 to date (Table 2; Supplementary Table S6). The physical locations of these MTAs were compared with the 72 MQTLs. Among the 72 MQTLs, 20 were co-located with at least one MTA from the GWAS. Most of these MQTLs (7/20) were co-located with only one MTA, while *MQTLLG7-2* (625 MTAs), *MQTLLG7-5* (47 MTAs), and *MQTLLG11-4* (98 MTAs) were co-located with multiple MTAs. Among the 20 GWAS-validated MQTLs, 11 were validated by only one of the six GWAS studies, five MQTLs (*MQTLLG6-1*, *MQTLLG6-3*, *MQTLLG6-5*, *MQTLLG7-5*, and *MQTLLG10-1*) were validated in two GWAS studies, three MQTLs (*MQTLLG7-2*, *MQTLLG8-2* and *MQTLLG11-4*) were validated in three GWAS studies, and one MQTL (*MQTLLG8-3*) was validated in four GWAS studies (Supplementary Table S6).

**TABLE 2:** Details of the GWAS used to validate the MQTL.

No	Source of genotype	Population size	Marker type/number	Number of MTA	Environment	Reference
1	Australian cultivated mungbean	466	SNP/16,462	9	Australia	Noble et al. (2018)
2	Chinese mungbean landraces	558	SNP/2,582,180	110	China	Han et al. (2022)
3	Chinese breeding lines, landraces, and non-Chinese lines	217	SNP/2,515,913	5,209	China	Liu et al. (2022a)
4	Wild and cultivated accessions	196	SNP/3,607,508	98	Thailand	Liu et al. (2022b)
5	USDA mung bean germplasm	484	SNP/26,550	87	USA	Chiteri et al. (2023)
6	Mungbean germplasm accessions from various origins	153	SNP/55,634	50	India	Manjunatha et al. (2023)

### Conserved genomic regions and orthologous MQTL among legume crops

**FIGURE 4:**
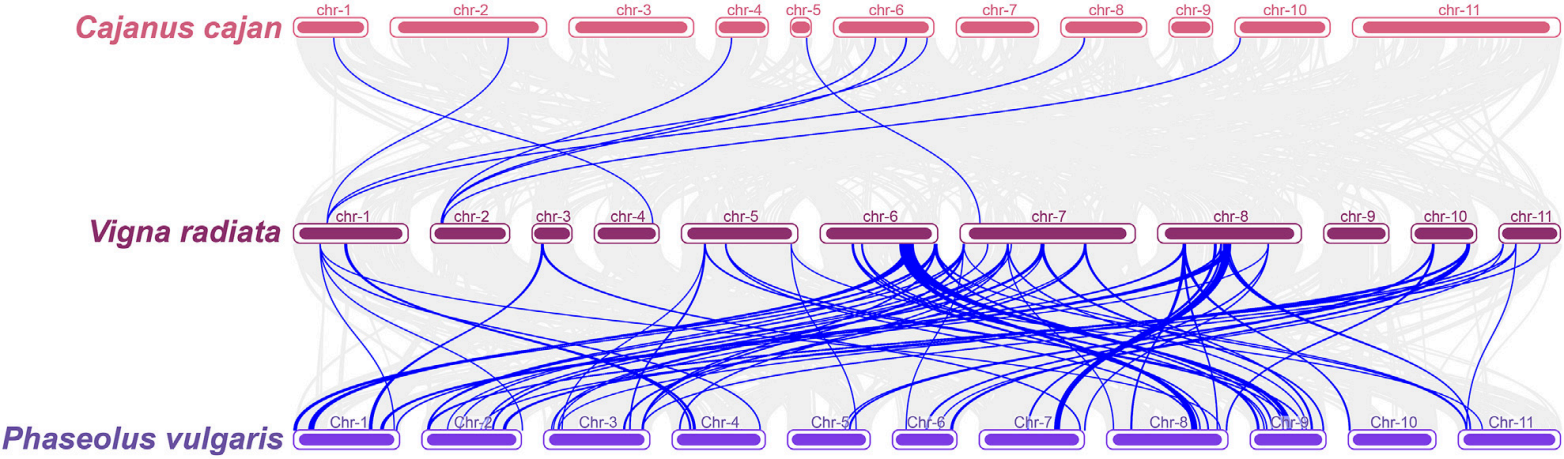
Colinearity analysis of *Vigna radiata* with two legume crops (*Cajanus cajan* and *Phaseolus vulgaris*). Gray lines indicate blocks of covariates between *Vigna radiata* and two other legume crops, and blue lines highlight homologous gene models within MQTL regions for the three legume crops.

Synteny analysis of the conserved genomic regions between mungbean, pigeonpea and common bean revealed that mungbean has 1,404 orthologs with pigeonpea and 1,504 orthologs with common bean (Supplementary Table S7). Among the 552 conserved genomic regions identified between mungbean and common bean, 15 common bean MQTLs were identified as orthologous MQTLs (OrMQTL) to 22 mungbean MQTLs. For example, the mungbean *MQTLLG8-3* was orthologous to two common bean MQTLs (*MQTL-YC7.5* and *MQTL-YC8.3*), and the *MQTLLG6-4* was isogenic to three common bean MQTLs (*MQTL YC1.2*, *MQTL-YC6.1* and *MQTL-YC8.3*). Additionally, the number of conserved gene models between OrMQTLs in mungbean and common bean ranged from 4 (*MQTLLG7-6*) to 108 (*MQTLLG6-4*), with 11 OrMQTLs containing at least 20 conserved gene models. Among the 33 MQTLs for agronomic traits, fertility restoration, and seed quality traits in pigeonpea, only 18 conserved gene models were identified in mungbean. This limited number was attributed to the small number of gene models within the MQTL regions, and no OrMQTLs were identified between mungbean and pigeonpea (Figure 4; Supplementary Table S7).

### Candidate gene mining based on orthologs within breeder’s MQTL regions

Twenty breeder’s MQTLs were screened for candidate gene mining in MQTL. These breeder’s MQTLs were associated with several mungbean yield and yield-related traits, indicating that key candidate genes controlling these traits might be located within these breeder’s MQTL regions (Supplementary Table S5). A total of 339 gene models were identified within these breeder’s MQTL regions, with the number of gene models per breeder’s MQTL ranged from 1 (*MQTLLG3-4*, *MQTLLG9-4*, and *MQTLLG9-5*) to 125 (*MQTLLG7-2*) (Supplementary Table S8). To further explore candidate genes affecting yield-related traits in mungbean, 22 mungbean orthologs of *Arabidopsis* and rice genes related to yield traits such as tiller number, starch synthesis, seed germination, and plant height were identified within the breeder’s MQTL regions through homology comparison with rice and *Arabidopsis*. Among these, 10 orthologs were derived from rice and 13 from *A. thaliana*, with one gene being identical to a rice gene (Supplementary Table S9).

## Discussion

### QTL meta-analysis reveals genetic architecture of yield and yield-related traits

Integrating 660 QTLs from diverse studies revealed genomic hotspots on LG2 and LG4 (Figure 2b), with over 14% of QTLs clustering on LG2. This uneven distribution aligns with synteny patterns in common bean (Izquierdo et al., 2023), suggesting conserved selection pressure on these regions. Notably, the preponderance of QTLs associated with growth period-related traits, pod-related traits, plant height, and grain weight highlights these as key determinants of yield potential (Figure 2a). This observation aligns with reported genetic control mechanisms in other legume crops (Izquierdo et al., 2023). These QTL were unevenly distributed across linkage groups (Figure 2b), suggests the existence of genomic hotspots with concentrated genetic potential for yield improvement in mungbean.

As the first QTL meta-analysis in mungbean, our study integrated seven published genetic maps to construct a high-density consensus genetic map (Supplementary Table S1). Through integration of seven published genetic maps, we constructed a high-density consensus genetic map (Supplementary Table S1), mapping most of the initial QTLs (590 out of 660) to this consensus map, and 72 MQTLs were identified via meta-analysis (Figures 1D,E). The statistical confidence of MQTLs correlates positively with the number of underlying component QTLs (Quraishi et al., 2017). Notably, 75% of our MQTLs incorporated ≥3 initial QTLs, with 58.3% (42/72) containing ≥6 component QTLs (Figure 3a), suggesting that these MQTLs have high reliability. Moreover, the average CI of MQTLs was reduced 6.26-fold compared to that of initial QTLs (Figure 3b). From these, we identified 20 breeder’s MQTLs characterized by high contributions (PVE >10%), narrow CIs (<2 cM), and more initial QTLs (≥4). Significantly, most of these breeder’s MQTLs exhibited pleiotropic effects on multiple yield-related traits, with three key loci (*MQTLLG2-5*, *MQTLLG4-6*, and *MQTLLG9-4*) influencing ≥10 yield-related traits (Supplementary Table S5), highlighting their potential for simultaneous improvement of multiple yield components.

### GWAS-MTA validation of MQTLs

GWAS based on linkage disequilibrium can detect minor-effect alleles that are missed in biparental populations. MTAs identified in GWAS can be used to validate candidate genes for QTL mapping (Han et al., 2018; Sallam et al., 2022). Using GWAS-MTA to validate the accuracy of MQTL results has been reported in QTL meta-analysis in legume crops such as soybean, pigeonpea, and common bean (Chen et al., 2021; Halladakeri et al., 2023; Izquierdo et al., 2023). In the present study, 27.8% (20/72) of the MQTL were validated in six GWAS studies published in recent years for yield and yield-related traits in mungbean (Supplementary Table S6). Of these validated MQTLs, the majority (7/20) showed overlap with a single MTA, while 55% (11/20) were supported by only one GWAS dataset. Notably, four critical MQTLs (*MQTLLG7-2*, *MQTLLG8-2*, *MQTLLG8-3*, and *MQTLLG11-4*) exhibited co-located with multiple MTAs across ≥3 GWAS studies (Supplementary Table S6), strongly suggesting that these genomic regions likely harbor key genetic determinants of yield and yield-related traits. These high-confidence loci (e.g., *MQTLLG7-2* and *MQTLLG8-3*) represent prime targets for MAS, particularly due to their pleiotropic effects on ≥10 traits (Supplementary Table S5).

### Conservation of OrMQTLs in other legume crops

Comparative synteny analysis revealed conserved OrMQTLs associated with agronomically important traits among legume crops including mungbean, pigeonpea, and common bean (Halladakeri et al., 2023; Izquierdo et al., 2023). Specifically, we identified 22 conserved OrMQTLs sharing synteny between mungbean and common bean genomes (Figure 4), suggesting strong evolutionary selection pressures to preserve these regions due to their functional importance in legume biology. These conserved loci harbor numerous uncharacterized genes in mungbean that represent promising targets for future functional studies. Characterizing genes within OrMQTLs could elucidate the genetic networks regulating yield and yield-related traits across legume species and provide insights into the molecular basis of trait evolution in legume crops. Furthermore, molecular markers derived from these syntenic regions hold potential for accelerating marker-assisted breeding strategies aimed at enhancing yield in mungbean (Saini et al., 2022).

### Candidate gene identification in breeder’s MQTL regions

As traditional model plants, *A. thaliana* and rice possess extensively characterized genomic resources, making synteny analysis between mungbean and these species a valuable strategy for identifying candidate genes associated with agronomically important traits (Gaut, 2002). To precisely map MQTLs to physical genomic positions, we applied the methodology of Prakash et al. (2022), which calculates physical coordinates using chromosomal genetic-to-physical length ratios, as direct alignment of MQTL-flanking markers to the mungbean reference genome was hindered by low sequence identity and alignment scores. Within the 20 breeder’s MQTL regions, we identified 339 gene models, including 22 mungbean orthologs of known regulators of tiller number, starch synthesis, seed germination, plant height, and yield in *Arabidopsis* and rice (Supplementary Table S9). Notably, the *MQTLLG7-2* region harbors *Vradi07g07390*, an ortholog of the rice amino acid transporter gene *OsLHT1* that critically influences plant growth and yield (Wang et al., 2019), and *Vradi07g07630*, which shares homology with the rice ABA receptor gene *OsPYL/RCAR10* implicated in seed germination (Kim et al., 2012). Furthermore, the *MQTLLG5-3* region contains *Vradi05g08600*, a functional ortholog of both the rice leaf morphology gene *YUCCA6* (Zhang et al., 2021), and and the Arabidopsis flowering time regulator *YUC8* (Ståldal et al., 2012), suggesting pleiotropic roles in yield-related traits. The findings not only advances our understanding of the genetic control of yield in mungbean but also provides a foundation for the development of molecular markers and the implementation of MAS in mungbean breeding programs.

## Conclusion

In conclusion, the integration of QTL meta-analysis, GWAS validation, orthologous MQTL, and comparative genomics has provided a comprehensive understanding of the genetic architecture underlying yield and yield-related traits in mungbean. This study identified a total of 72 MQTLs, with the average CI being narrowed down by 6.26-fold compared to the initial QTLs. Among these, 20 MQTLs were validated through GWAS-MTAs, while 22 OrMQTLs were detected across different legume crops through colinearity analysis. Furthermore, 22 mungbean orthologs of yield and yield-related genes from rice and *Arabidopsis* were identified in the breeder’s MQTL regions using a comparative genomics approach. These findings significantly enhance our understanding of the genetic mechanisms governing yield and yield-related traits in mungbean, providing valuable insights for future breeding programs.

## Data Availability

The original contributions presented in the study are included in the article/Supplementary Material, further inquiries can be directed to the corresponding authors.
