# Supplementary material for: Genetic dissection of yield and yield-related traits in mungbean based on QTL meta-analysis
Source: Front Genet. 2025 May 8;16:1600979. doi: 10.3389/fgene.2025.1600979 (PMC12095261; doi:10.3389/fgene.2025.1600979)

**Supplementary Data S1. R package LPmerge Script.**

library(LPmerge)

library(Rglpk)

setwd("C:/Users/Du/Desktop/mungbean/")

map.names = c("H", "I", "W1", "L", "W2", "W3", "Y")

#############1

map.names = c("H", "I", "W1", "L", "W2", "W3", "Y")##1

Maps <- list()

i <- 1

for (i in 1:7) {

filename <- paste(map.names[i],".csv",sep="")

input <- read.csv(filename,header=T,as.is=T,check.names=F)

Maps[[i]] <- input[which(input$chr=="8"), c(1,3)]

}

names(Maps) <- map.names

str(Maps)

print(link.map.lengths <- unlist(lapply(Maps,function(x){max(x$cM)})))

mean(link.map.lengths)

unweighted <- LPmerge(Maps,max.interval=1:4)

head(unweighted[[1]])

write.csv(unweighted[[1]], file = "CA_8.txt")

sink("out_CA_8.txt")

print(input$marker)

print(input$chr)

print(input$cM)

print("link.map.lengths results")

print("map.names results")

print("unweighted <- LPmerge(Maps,max.interval=1:4)")

print(unweighted)

sink()

**Supplementary Data S2. The optimal number of MQTL per chromosome was determined based on the model selection criteria (_model.txt) as well as the 95% confidence intervals (CIs) and peak locations of the MQTL (_table.txt).**

LG1

_model.txt


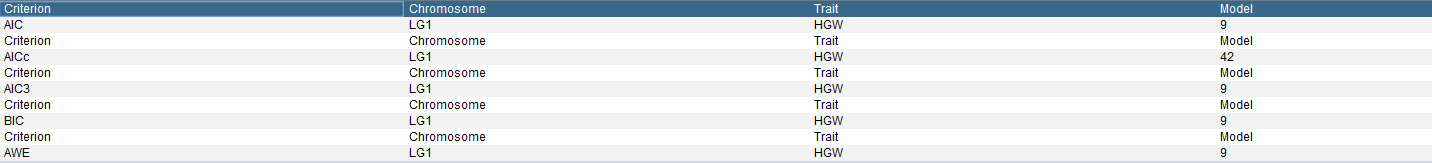


_table.txt


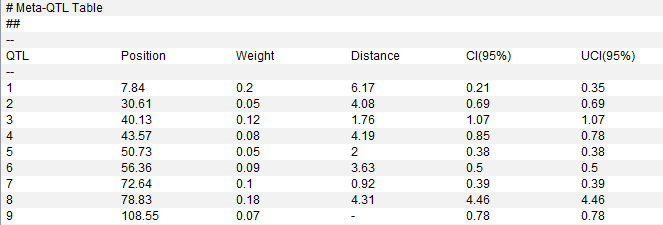


LG2

_model.txt


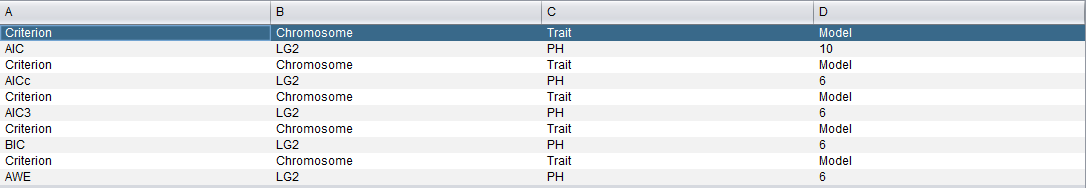


_table.txt


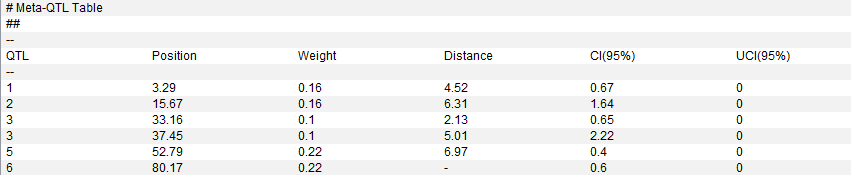


LG3

_model.txt


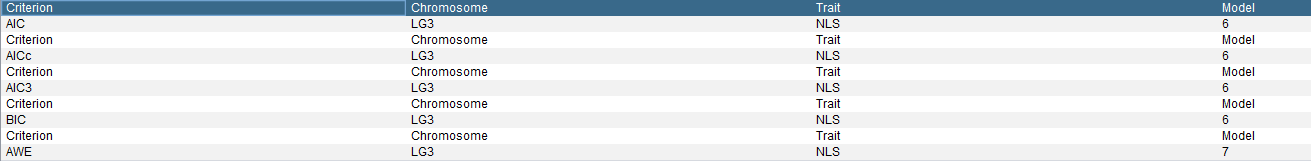


_table.txt


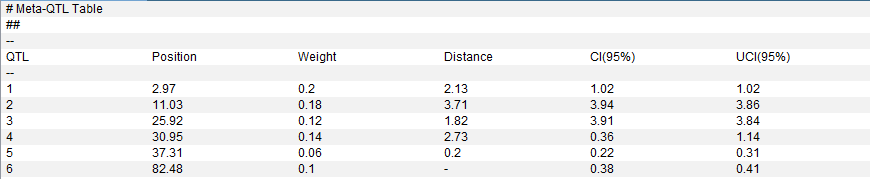


LG4

_model.txt


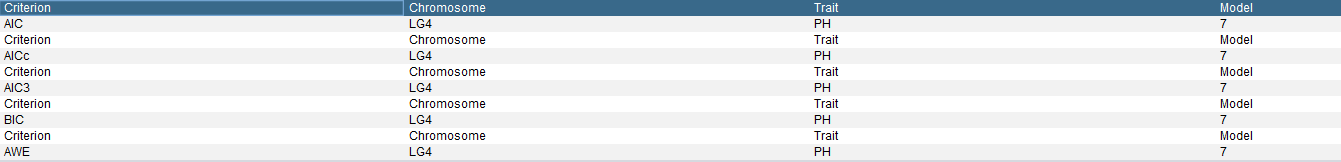


_table.txt


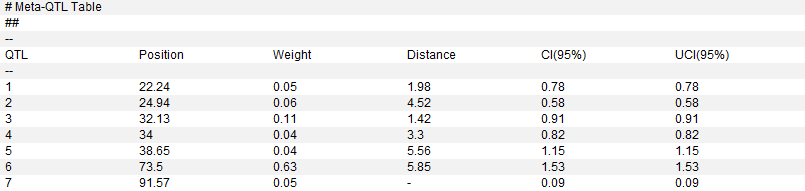


LG5

_model.txt


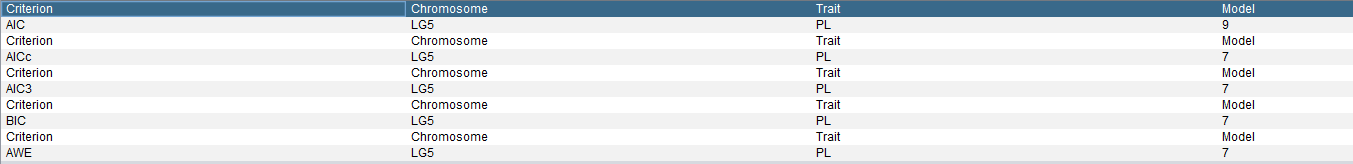


_table.txt


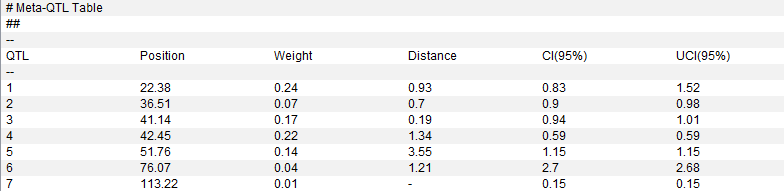


LG6

_model.txt


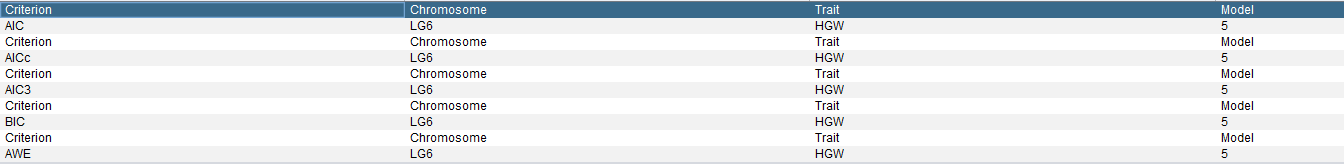


_table.txt


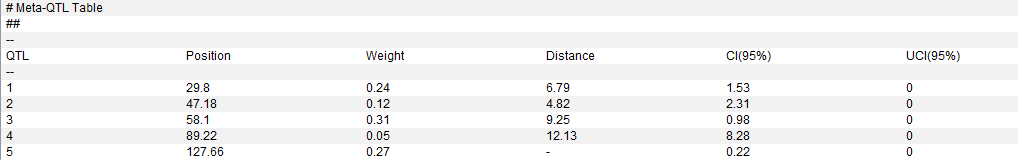


LG7

_model.txt


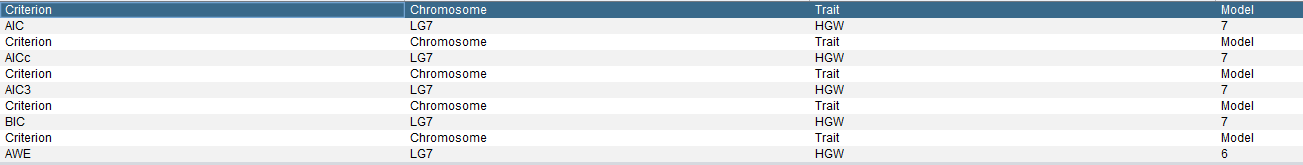


_table.txt


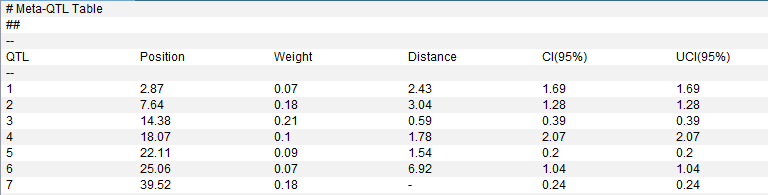


LG8

_model.txt


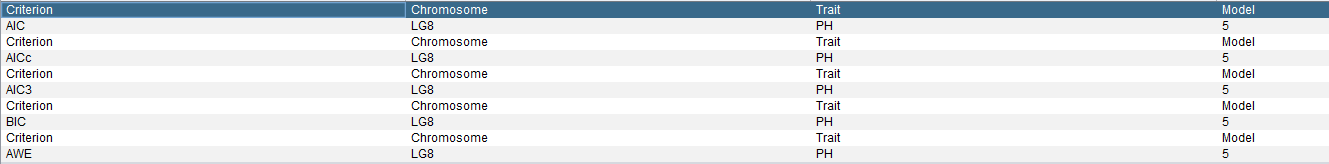


_table.txt


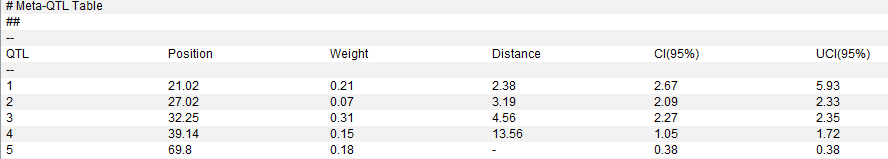


LG9

_model.txt


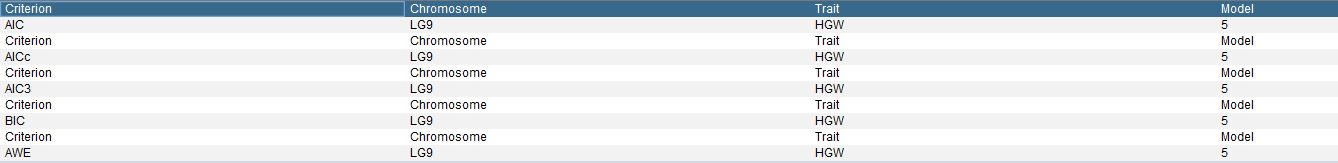


_table.txt


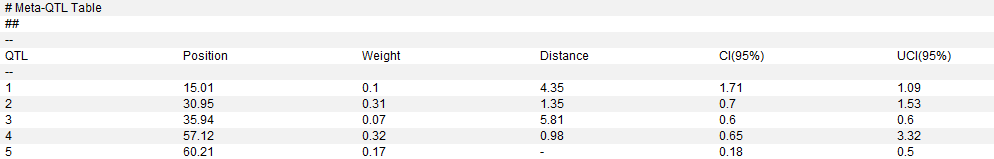


LG10

_model.txt


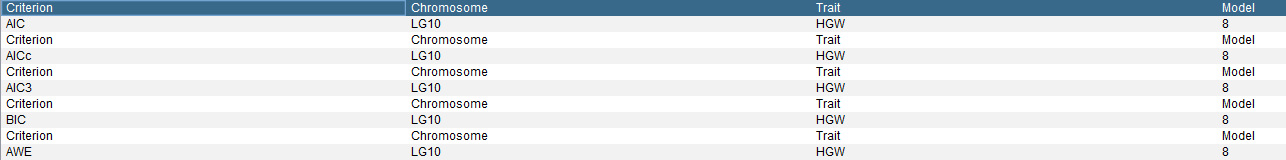


_table.txt


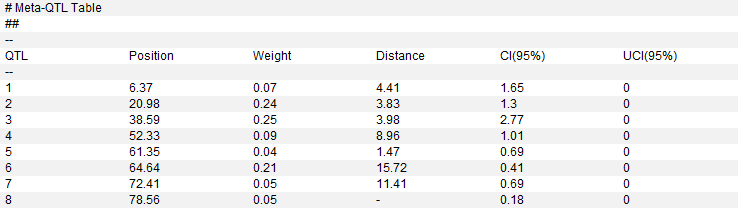


LG11

_model.txt


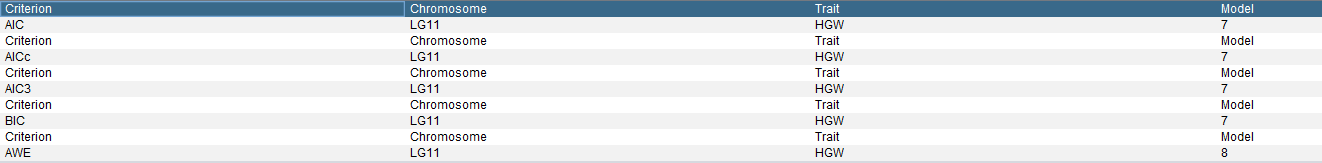


_table.txt


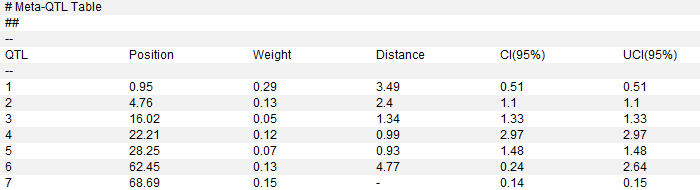

Supplement: Supplementary file 2 [file DataSheet1.docx]
